# Supplementary material for: Checkpoint Kinase 1 Inhibitor Combined with Low Dose Hydroxyurea Promotes ATM-Activated NF-κB-Dependent Pro-Inflammatory Chemokine Expression in Melanomas
Source: Cancers (Basel). 2025 May 29;17(11):1817. doi: 10.3390/cancers17111817 (PMC12153532; doi:10.3390/cancers17111817)
Supplement: Supplementary file 1 [file cancers-17-01817-s001.zip › Supplementary Data text.pdf]

Supplementary Data

Supplementary Table S1. RT-qPCR Oligonucleotide KiCqStart Primers (MERCK, Sigma-Aldrich)

Target Gene	Direction	Sequence	Oligo #
TGFB1	Forward	AACCCACAACGAAATCTATG	3033018775-60/0
	Reverse	CTTTTAACTTGAGCCTCAGC	3033018775-60/1
CCL2	Forward	AGACTAACCCAGAAACATCC	3033018775-70/0
	Reverse	ATTGATTGCATCTGGCTG	3033018775-70/1
CCL5	Forward	ACTTGCCTCCCCATATTC	3034005936-30/0
	Reverse	AAGAGTTGATGTACTCCCG	3034005936-30/01
CXCL10	Forward	AAAGCAGTTAGCAAGGAAAG	3033018775-90/0
	Reverse	TCATTGGTCACCTTTTAGTG	3033018775-90/1
XCL1	Forward	TACATTGTGGAAGGTGTAGG	3033018775-110/0
	Reverse	TGGTGTAGGTCTTGATTCTG	3033018775-110/1
VEGFA	Forward	AATGTGAATGCAGACCAAAG	3033018775-120/0
	Reverse	GACTTATACCGGGATTTCTTG	3033018775-120/1
CXCL9	Forward	AGGTCAGCCAAAAGAAAAAG	3033018775-130/0
	Reverse	TGAAGTGGTCTCTTATGTAGTC	3033018775-130/1
IL-6	Forward	GCAGAAAAAGGCAAAGAATC	3033572500-60/0
	Reverse	CTACATTTGCCGAAGAGC	3033572500-60/1
IL-8	Forward	GTTTTTGAAGAGGGCTGAG	3033572500-70/0
	Reverse	TTTGCTTGAAGTTTCACTGG	3033572500-70/1
TNF	Forward	AGGCAGTCAGATCATCTTC	3033869053-10/0
	Reverse	TTATCTCTCAGCTCCACG	3033869053-10/1
YWHAZ	Forward	AACCTTGACATTGTGGACATC	3033018775-50/0
	Reverse	AAAACTATTTGTGGGACAGC	3033018775-50/1

Supplementary Table S2. Known mutation status of the melanoma cell lines

Cell Lines	BRAF	CDKN2A	CDK4	Nras	p14ARF	PIK3CA	PTEN		p53
A2058	**V599E**	wt	wt	wt	wt		**L112Q, L186M**	**homo,het**	wt
BL	**V599E**	**E88K**	wt	wt	**G102E**		**Q298Stop**	**homo**	**I195T**
D04	wt	**HD**	wt	**Q61L**	HD		wt		wt
D25	**V599E**	wt	wt	wt	wt	wt	wt		**D281N**
MM329	wt	wt	wt	wt	wt	**I139M**	wt		wt
SKMEL13	**V599E**	**HD**	wt	wt	**HD**	wt	wt		**R248W**
SKMEL28	**V599E**	wt	**R24C**	wt	wt		**T167A**	**Homo**	**L145R**

Supplementary Figures

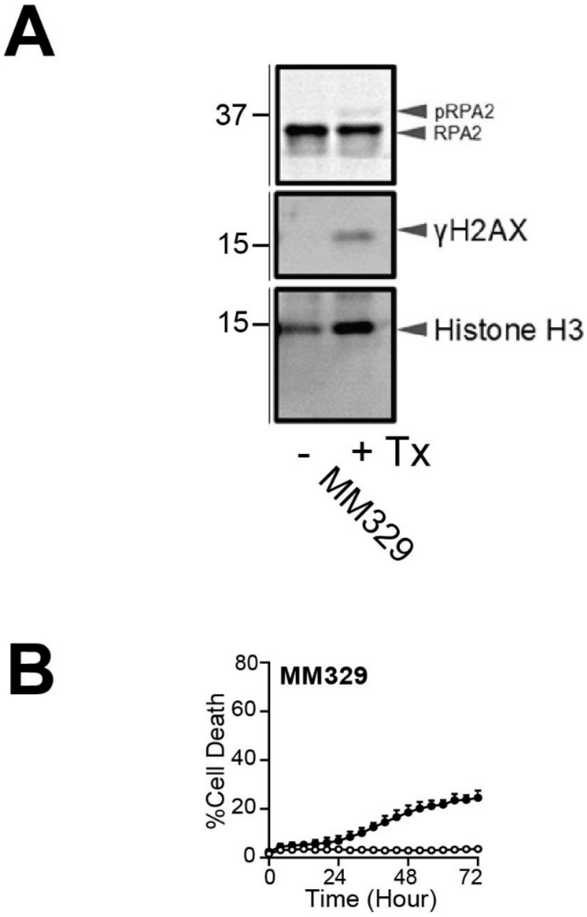

Supplementary Figure S1: SRA737+LDHU induces replication stress and DNA damage. A: MM329 cells were treated with or without 1μM SRA737 + 0.2mM HU and harvested at 24 hours. Cell lysates were immunoblotted for γH2AX as a marker for DNA damage, and RPA2 for replication stress. Histone H3 was used as a loading control. B: MM329 cells were treated as in A and cells followed using Incucyte live cell imaging using Sytox Green as a marker of cell death. The % cell death is shown. The data are the mean and SD from three replicates.

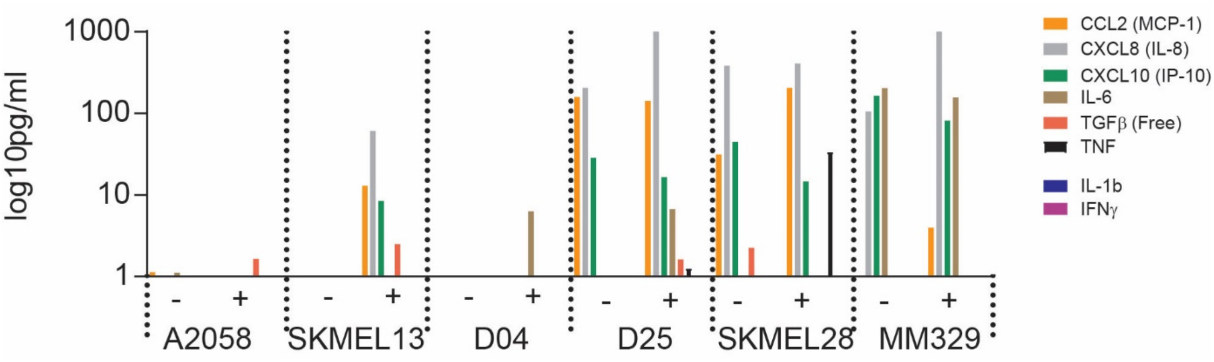

Supplementary Figure S2: SRA737+LDHU increases release of cytokines and chemokines from human melanoma cell lines. Cells were treated without or with (-, +) 1μM SRA737 + 0.2mM HU. Cell culture media supernatants from melanoma cell lines were harvested 48 hours after treatment. Cytokine/chemokine levels were measured using LegendPlex bead array and flow cytometry. Data represents the mean of duplicate wells and two independent experiments.

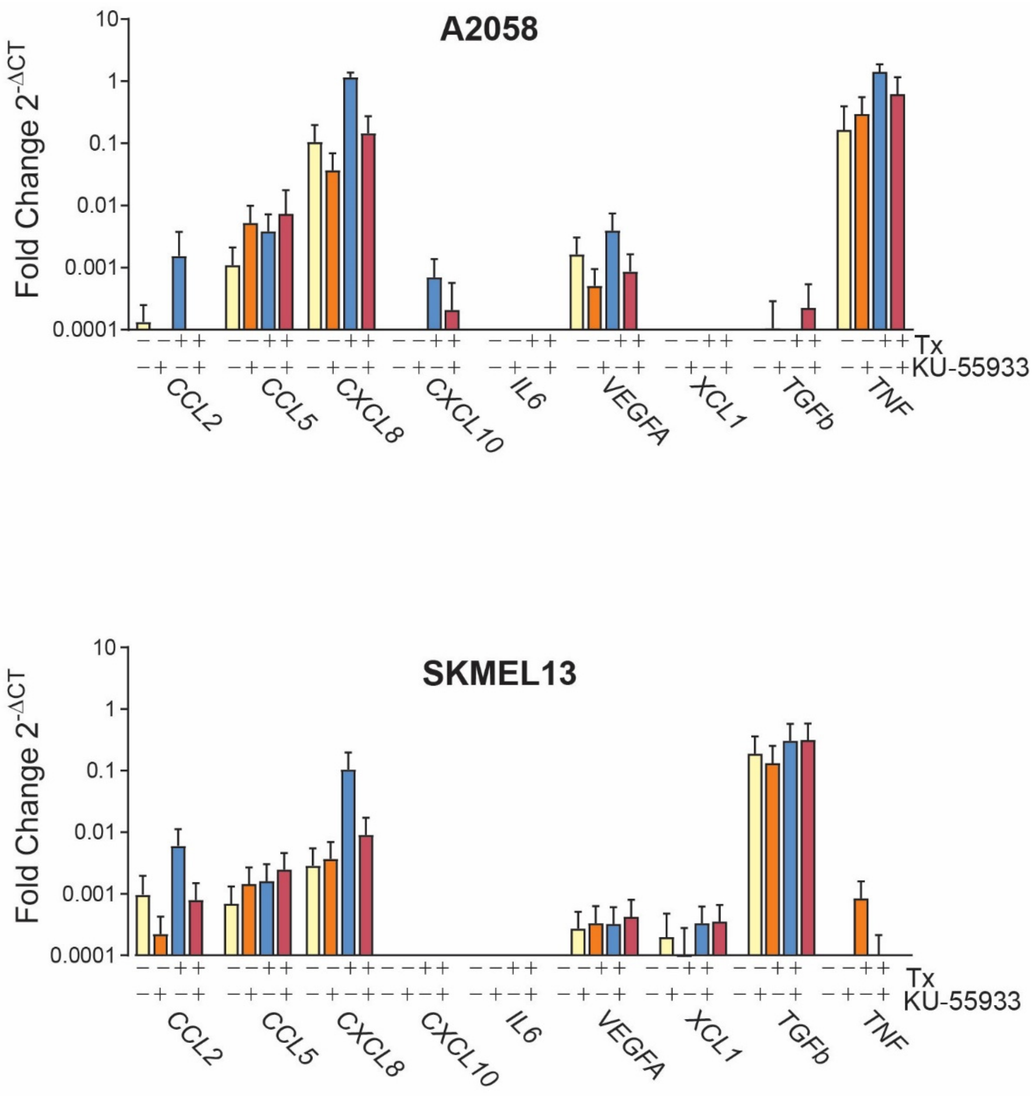

Supplementary Figure S3: Inhibition of ATM with SRA737+LDHU returns expression of NF-κB-target cytokine/chemokine genes to basal levels. A2058 and SKMEL13 were treated with ATM inhibitor 10µM KU-55933 in addition to 1µM SRA737 + 0.2mM HU for 24 hours and harvested for RNA extraction, followed by RT-qPCR. Relative expression (ΔCt) values were normalized against the housekeeping gene YWHAZ, then log transformed ($2^{-\Delta Ct}$) for visual representation as fold change on a log10 y-axis. Data represents the mean with standard deviation error bars and is representative of 3 independent experiments (n=3), taking the mean of triplicate wells for each experiment. Two-way ANOVA was performed on ΔCt values with multiple comparisons of biological replicate means of each treatment group. P-values were corrected using the Tukey test with a 0.05 (95% CI) threshold.
