# Supplementary figures and images for: Checkpoint Kinase 1 Inhibitor Combined with Low Dose Hydroxyurea Promotes ATM-Activated NF-κB-Dependent Pro-Inflammatory Chemokine Expression in Melanomas
Source: Cancers (Basel). 2025 May 29;17(11):1817. doi: 10.3390/cancers17111817 (PMC12153532; doi:10.3390/cancers17111817)

Figure 1A

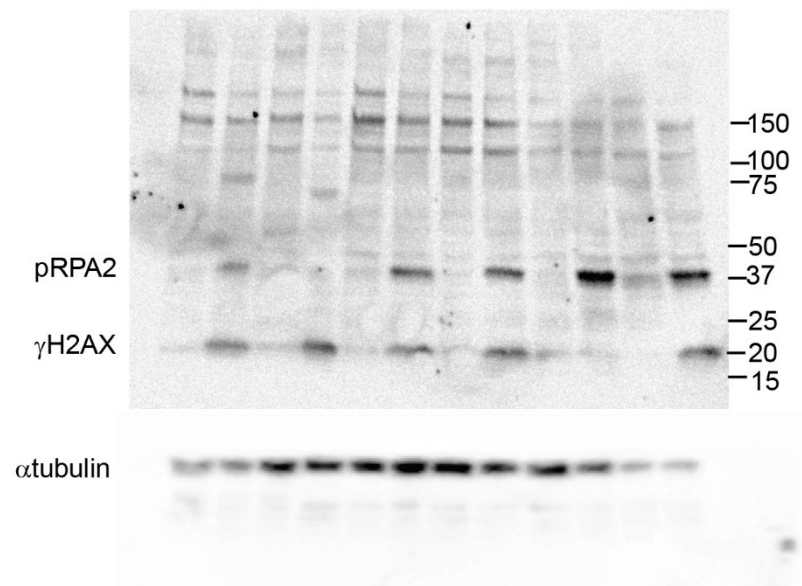

Figure 5 A

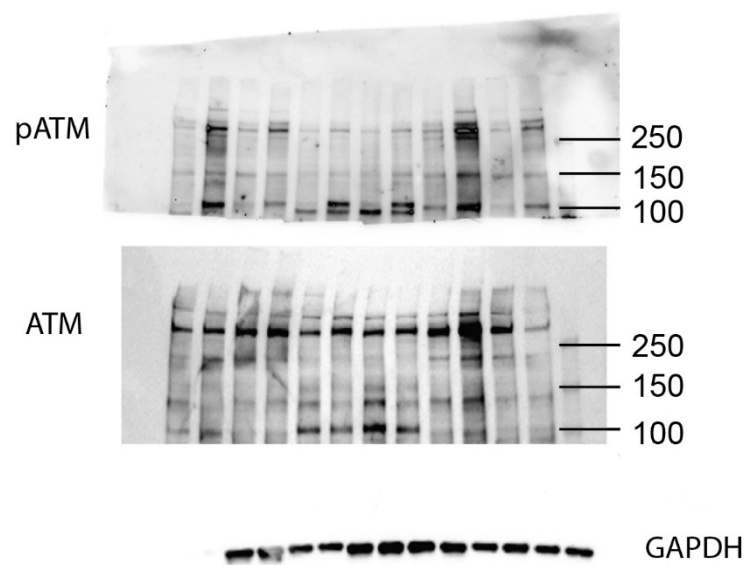

Figure 5 B

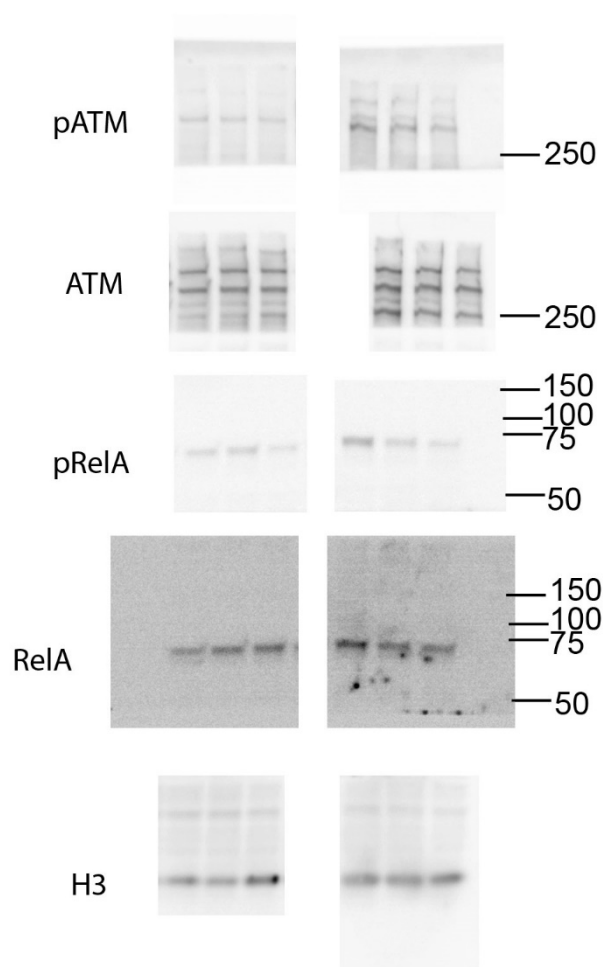

Supplementary Figure S1

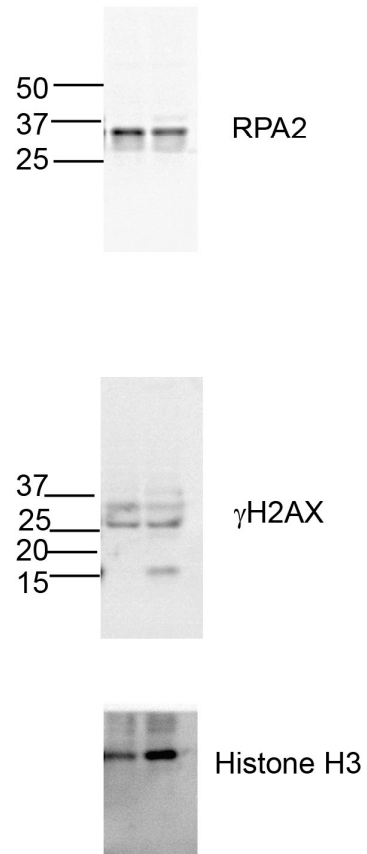

Supplement: Supplementary file 1 [file cancers-17-01817-s001.zip › File S1.pdf]
